# Evaluating the Autonomy of the *Drosophila* Circadian Clock in Dissociated Neuronal Culture

**DOI:** 10.3389/fncel.2017.00317

**Published:** 2017-10-12

**Authors:** Virginie Sabado, Ludovic Vienne, Emi Nagoshi

**Affiliations:** Department of Genetics and Evolution, Sciences III, University of Geneva, Geneva, Switzerland

**Keywords:** *Drosophila*, circadian rhythms, time-lapse imaging, non-cell autonomous, dispersed culture, fluorescent circadian reporter

## Abstract

Circadian behavioral rhythms offer an excellent model to study intricate interactions between the molecular and neuronal mechanisms of behavior. In mammals, pacemaker neurons in the suprachiasmatic nucleus (SCN) generate rhythms cell-autonomously, which are synchronized by the network interactions within the circadian circuit to drive behavioral rhythms. However, whether this principle is universal to circadian systems in animals remains unanswered. Here, we examined the autonomy of the *Drosophila* circadian clock by monitoring transcriptional and post-transcriptional rhythms of individual clock neurons in dispersed culture with time-lapse microscopy. Expression patterns of the transcriptional reporter show that CLOCK/CYCLE (CLK/CYC)-mediated transcription is constantly active in dissociated clock neurons. In contrast, the expression profile of the post-transcriptional reporter indicates that PERIOD (PER) protein levels fluctuate and ~10% of cells display rhythms in PER levels with periods in the circadian range. Nevertheless, PER and TIM are enriched in the cytoplasm and no periodic PER nuclear accumulation was observed. These results suggest that repression of CLK/CYC-mediated transcription by nuclear PER is impaired, and thus the negative feedback loop of the molecular clock is incomplete in isolated clock neurons. We further demonstrate that, by pharmacological assays using the non-amidated form of neuropeptide pigment-dispersing factor (PDF), which could be specifically secreted from larval LNvs and adult s-LNvs, downstream events of the PDF signaling are partly impaired in dissociated larval clock neurons. Although non-amidated PDF is likely to be less active than the amidated one, these results point out the possibility that alteration in PDF downstream signaling may play a role in dampening of molecular rhythms in isolated clock neurons. Taken together, our results suggest that *Drosophila* clocks are weak oscillators that need to be in the intact circadian circuit to generate robust 24-h rhythms.

## Introduction

Virtually all organisms have circadian clocks, which emerged as an adaptation strategy for life on Earth. Circadian rhythms are synchronized (entrained) to environmental cues, e.g., light-dark cycles (LD) and warm-cool cycles, thereby allowing organisms to anticipate and prepare for daily and seasonal changes. A fundamental property of circadian rhythms is their persistence in the absence of inputs, which indicates the existence of an endogenous clock that can free-run with a period of ~24 h (Zhang and Emery, 2012; Granados-Fuentes and Herzog, 2013).

Some unicellular organisms exhibit circadian rhythms autonomously (Webb and Oates, 2016). In multicellular organisms, clock cells are found in many tissues, where rhythms are synchronized directly by external cues or coordinated by a central clock, such as the SCN in mammals. Different studies using reporters of the molecular clock have shown that circadian rhythmicity is maintained in individual SCN neurons in organotypic culture (Yamaguchi et al., 2003; Yoo et al., 2004; Fang et al., 2007; Liu et al., 2007). In dissociated SCN culture, high-amplitude circadian rhythms persist in most but not all neurons (Liu et al., 2007). The proportion of robustly cycling neurons further decreases and the rhythms are more desynchronized in low-density culture (Webb et al., 2009). It has been concluded that, although fundamentally cell-autonomous, SCN neurons require intercellular coupling to exhibit robust circadian rhythms (Ko et al., 2010). This is in stark contrast to mammalian fibroblasts, which generate robust, self-sustaining rhythms regardless of density and culture conditions (Nagoshi et al., 2004; Welsh et al., 2004). Thus, central clocks are less robust than peripheral clocks at the single cell level in mammals. Importantly, however, damped oscillators respond more readily to each other and to the environmental cycle to achieve fast synchronization (Ko et al., 2010; Webb and Oates, 2016). These characteristics are particularly advantageous for the central pacemaker.

In adult *Drosophila*, ~150 clock neurons form a circuit that orchestrates the circadian behavior. The circadian circuitry exists also in the larval brain, which is composed of three groups of clock neurons, i.e., five ventral Lateral Neurons (LNvs), two Dorsal Neurons 1 (DN1s) and two Dorsal Neurons 2 (DN2s) (reviewed in Helfrich-Forster, 2005). Larval circuitry is known to govern circadian behavior in response to light (Malpel et al., 2004; Mazzoni et al., 2005). In the core feedback loop of each clock neuron, the transcription of *period* (*per*) and *timeless* (*tim*) is activated by CLK/CYC heterodimer through binding to E-boxes located on the promoters of *per* and *tim* (Hardin and Panda, 2013). PER and TIM enter the nucleus and inhibit CLK/CYC activity, leading to the repression of their own transcription. Various post-transcriptional and post-translational regulations, such as PER phosphorylation and TIM degradation, take place to complete the core feedback loop in circa 24 h (Sheeba et al., 2008; Zhang and Emery, 2012). Light, the main input of the circadian clock, is sensed by photoreceptors and the blue-light photopigment cryptochrome (CRY; Reviewed in Yoshii et al., 2016). Light-activated CRY induces the degradation of TIM and destabilizes PER/TIM heterodimer, leading to the phase resetting or arrhythmicity depending on the light regime (Emery et al., 1998; Stanewsky et al., 1998; Zhang and Emery, 2012). Additional transcriptional feedback loops contribute to the robustness of the core feedback loop. Altogether, these interlocked Transcriptional-Translational Feedback Loops (TTFL) constitute the molecular clock (Sheeba et al., 2008; Hardin, 2011).

To examine the intrinsic properties of *Drosophila* clock neurons, we recently developed two fluorescent reporters of the molecular clockwork that permit single-cell recording of the transcriptional and post-transcriptional machineries (Sabado et al., 2017). The transcriptional reporter 3 × 69-VNP expresses the short-lived yellow fluorescent protein (VNP; VENUS-NLS-PEST) under the control of three tandem repeats of the E-box-containing 69-bp clock regulatory sequence (CRS) of *per* (Hao et al., 1997, 1999). 3 × 69-VNP thus mimics the CLK/CYC-dependent transcriptional rhythms and *per* mRNA oscillations. PER protein reporter PER-TDT was built through the fusion of *per* endogenous promoter, two-thirds of the coding sequence of *per*, the cDNA encoding the red fluorescent protein tandem TOMATO (TDT) and *per* 3′-UTR. Both reporters are expressed rhythmically in clock neurons *in vivo* and in brain explant culture with expected phases (Sabado et al., 2017).

In this study, we investigate the state of autonomy of isolated *Drosophila* clock neurons by taking advantage of the long-term live imaging of molecular clockwork with our fluorescent reporters. The results demonstrate that *Drosophila* clocks are intrinsically unstable oscillators that rely on intact circuitry to generate robust circadian rhythms.

## Results

**Figure 1:**
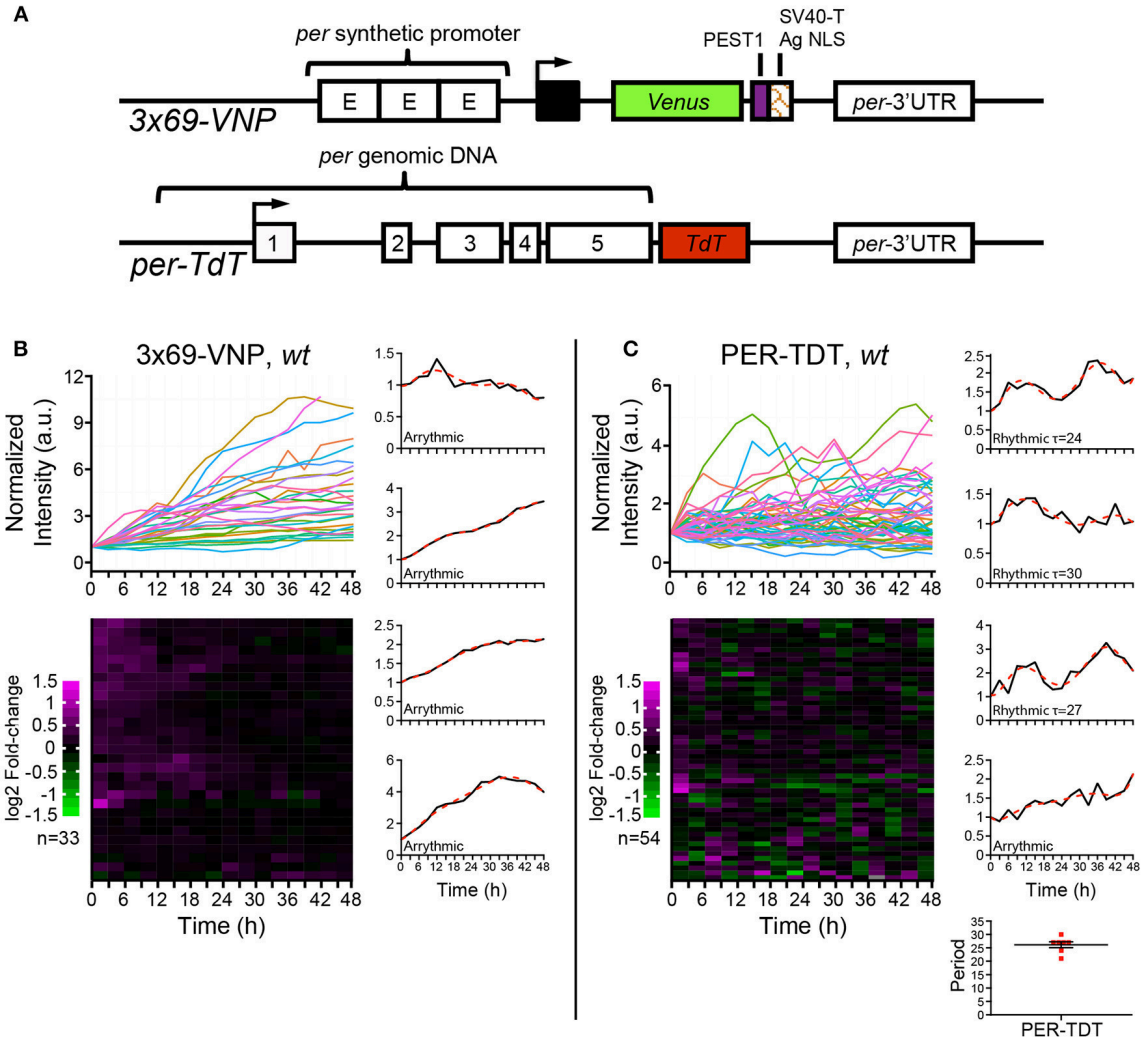
*Drosophila* neuronal clocks are poorly rhythmic in dissociated culture. **(A)** Schemes of the 3 × 69-VNP and PER-TDT reporters (Sabado et al., 2017). **(B)** 3 × 69-VNP and **(C)** PER-TDT expression in clock neurons in *wild-type* (*wt*) dissociated culture. Graphs indicate relative fluorescence intensity of individual cells normalized to the value at *t* = 0. Raster plots indicate the time course of fluorescence intensity fold-change in log2. Each row represents a single cell. Representative single cell traces are shown with the 6th order polynomial trend line (red dotted line). Note that PER-TDT profiles are similar between the LNvs labeled by *gal1118*>*mCD8::Venus* and non-LNv clock neurons, the expression profiles of both cell types are shown without distinction in **(B)**. Bottom right, periods of the 13% rhythmic neurons in PER-TDT expressing neurons. Mean period ± SEM was 26.143 ± 1.079 h. *n*, number of cells.

Previous works have shown that *Drosophila* clock neurons in cultured adult brains exhibit circadian molecular rhythms (Sellix et al., 2010; Roberts et al., 2015). By using fluorescent transcriptional reporter 3 × 69-VNP and PER protein reporter PER-TDT (Figure 1A, Supplementary Figures 1A,B), we have also demonstrated that molecular rhythms of larval clock neurons in cultured brains are detectable by time-lapse microscopy. Although reporter fluorescence levels varied between animals and among individual neurons, we observed circadian expression of both 3 × 69-VNP and PER-TDT in more than 50% of larval clock neurons (Sabado et al., 2017). These findings unequivocally indicate the ability of individual fly clock neurons to generate rhythms without environmental cues in the isolated brain, where circadian circuit is intact. However, whether fly clock neurons are cell-autonomous oscillators remains to be formally tested.

To this end, we cultured dissociated neurons derived from third instar larval brains in the same medium used for brain explant culture and imaged 3 × 69-VNP and PER-TDT reporter expression by time-lapse microscopy. Clock neurons expressing 3 × 69-VNP or PER-TDT were visualized by co-expressing *UAS-mCD8::RFP* with *1982clk-gal4* (labeling all three subtypes), or *UAS-mCD8::Venus* with *gal1118* (labeling the LNvs), respectively. Images were acquired every 3 h for 48 h, i.e., with the same interval and duration of time-lapse used to monitor rhythms in cultured brains (Sabado et al., 2017). Surprisingly, the majority of clock neurons displayed a steady increase in 3 × 69-VNP levels over time without detectable oscillations. By contrast, PER-TDT expression showed fluctuations of varying patterns with ~13% of the clock neurons displaying rhythms in the circadian range (mean period ± SEM, 26.143 ± 1.079 h; Figures 1B,C, Supplementary Movies S1, S2). PER-TDT expression profiles of the LNvs and non-LNvs were indistinguishable, and the rhythmic cells were not restricted to the LNvs.

**Figure 2:**
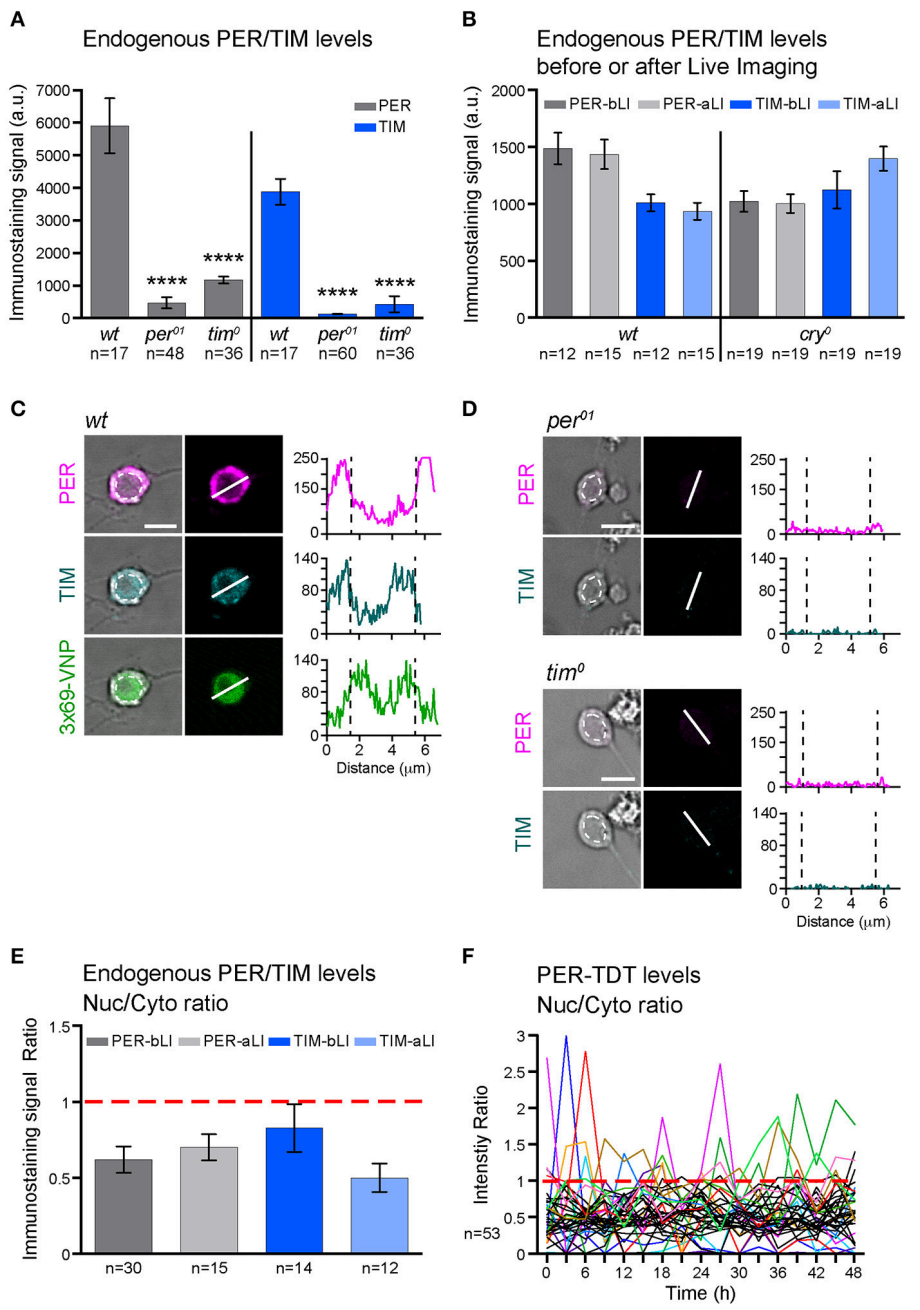
PER and TIM are mainly cytoplasmic in dissociated cultured neurons. **(A–E)** The levels and subcellular localization of endogenous PER and TIM were analyzed by immunostaining. **(A)** *wt* clock neurons express PER and TIM (^****^p < 0.0001 between *wt* and *per*^*01*^ or *tim*^*0*^ by one-way ANOVA with Tukey's correction for multiple comparisons). **(B)** PER and TIM expression levels did not decrease after live imaging. bLI, before live imaging; aLI, after live imaging. Mean, error bars represent ± SEM. The values between before and after live imaging were compared by one-way ANOVA with Tukey's correction for multiple comparisons and showed no significant differences. **(C,D)** Representative images of cultured neurons double stained for PER and TIM. Scale bar, 5 μm. **(C)** Clock neurons expressing 3 × 69-VNP also express PER and TIM. The graphs represent the z-axis profiles of the fluorescence along the indicated line. Dotted lines in the graphs indicate the position of the nucleus, which was manually defined on the bright-field image (dotted circles on the left panels). Y-axes represent the fluorescence intensity in arbitrary unit. Z-axis profiles are plotted for the presentation purpose only but not for quantitative analysis. **(D)** Negative controls for anti-PER/TIM staining performed on the neuron derived from *per01* or tim0 mutant larvae. **(E)** Nuclear/Cytoplasmic (Nuc/Cyto) ratio of PER and TIM staining signal before and after live imaging. Fluorescence intensity was measured using 3D image analysis. Mean ± SEM. Comparison between aLI and bLI by Mann-Whitney test showed no significant differences. **(F)** Nuc/Cyto ratio of PER-TDT fluorescence intensity of individual clock neurons during 48-h live imaging of *wt* clock neurons. For clarity, only the cells that showed nuclear enrichment (ratio > 1) are shown in color.

The cultured larval neurons maintained the characteristics of healthy primary neurons (Saad et al., 2012) throughout time-lapse imaging (Supplementary Figure 2). The neurons expressing reporters in dissociated culture also expressed endogenous PER and TIM; their levels did not decrease after live imaging and were not significantly different between *wild*-*type* (*wt*) and *cry*^*0*^ backgrounds (Figures 2A–D). Therefore, the absence of 3 × 69-VNP rhythms in dissociated clock neurons is not due to the deterioration of viability, laser illumination or light-mediated activation of CRY but rather reflects the properties of neuronal oscillators. The observation that PER-TDT levels do not linearly increase but fluctuate suggests that certain post-transcriptional regulations contributing to the generation of PER rhythms is still operational in isolated neurons, preventing the over-accumulation of PER despite the constant upregulation of CLK/CYC-mediated transcription. In addition, some transcriptional regulation of *per* via cis-regulatory elements other than CRS is probably also functional in cultured clock neurons, contributing to the fluctuating PER-TDT expression.

To explore the mechanisms leading to these changes in molecular rhythms in isolated neurons, we next analyzed the subcellular localization of endogenous PER and TIM. Interestingly, PER and TIM were detected in both nucleus and cytoplasm but enriched in the cytoplasm of cultured clock neurons. Live imaging did not affect the nuclear/cytoplasmic ratio (Figures 2C,E). Furthermore, consistent with the subcellular localization pattern of endogenous PER, PER-TDT reporter was enriched in the cytoplasm throughout the recording period of live imaging. A small proportion of neurons showed PER-TDT nuclear enrichment sporadically, but without any apparent rhythms (Figure 2F). These results indicate that timed nuclear entry of PER is impaired in dissociated cultured neurons. This finding further suggests that the feedback inhibition of CLK/CYC transcriptional activity by PER may be dysregulated, resulting in a continuous, non-rhythmic CLK/CYC-mediated transcription. This would explain the constant rise of 3 × 69-VNP signals in dissociated clock neurons.

Because clock neurons can generate both 3 × 69-VNP and PER-TDT rhythms in whole brain culture (Sabado et al., 2017), the disruption of molecular rhythms in dissociated culture may be caused by the lack of intercellular interactions that normally occur *in vivo* and in brain explants. Network interactions within the circadian circuit are mediated by various neuropeptides and neurotransmitter signaling (Beckwith and Ceriani, 2015). In particular, a number of studies have shown that the neuropeptide pigment-dispersing factor (PDF) is critical for synchronizing and maintaining rhythms of clock neurons (Peng et al., 2003; Helfrich-Forster, 2005; Yoshii et al., 2009; Yao and Shafer, 2014; Beckwith and Ceriani, 2015). Recent works also reported that PDF regulates the phase of neuronal activity rhythms of different clock neuron subgroups (Liang et al., 2016, 2017). We also recently showed that PDF has a nighttime-specific dual role in regulating the molecular clockwork: enhancing CLK/CYC-mediated transcription and stabilizing PER in larval brains. We further demonstrated that the addition of PDF in dissociated culture increases 3 × 69-VNP signals in clock neurons, which indicates that PDF signaling stimulates CLK/CYC-mediated transcription cell-autonomously (Sabado et al., 2017). However, whether PDF affects PER stability cell-autonomously in dissociated neurons has not been examined.

**Figure 3:**
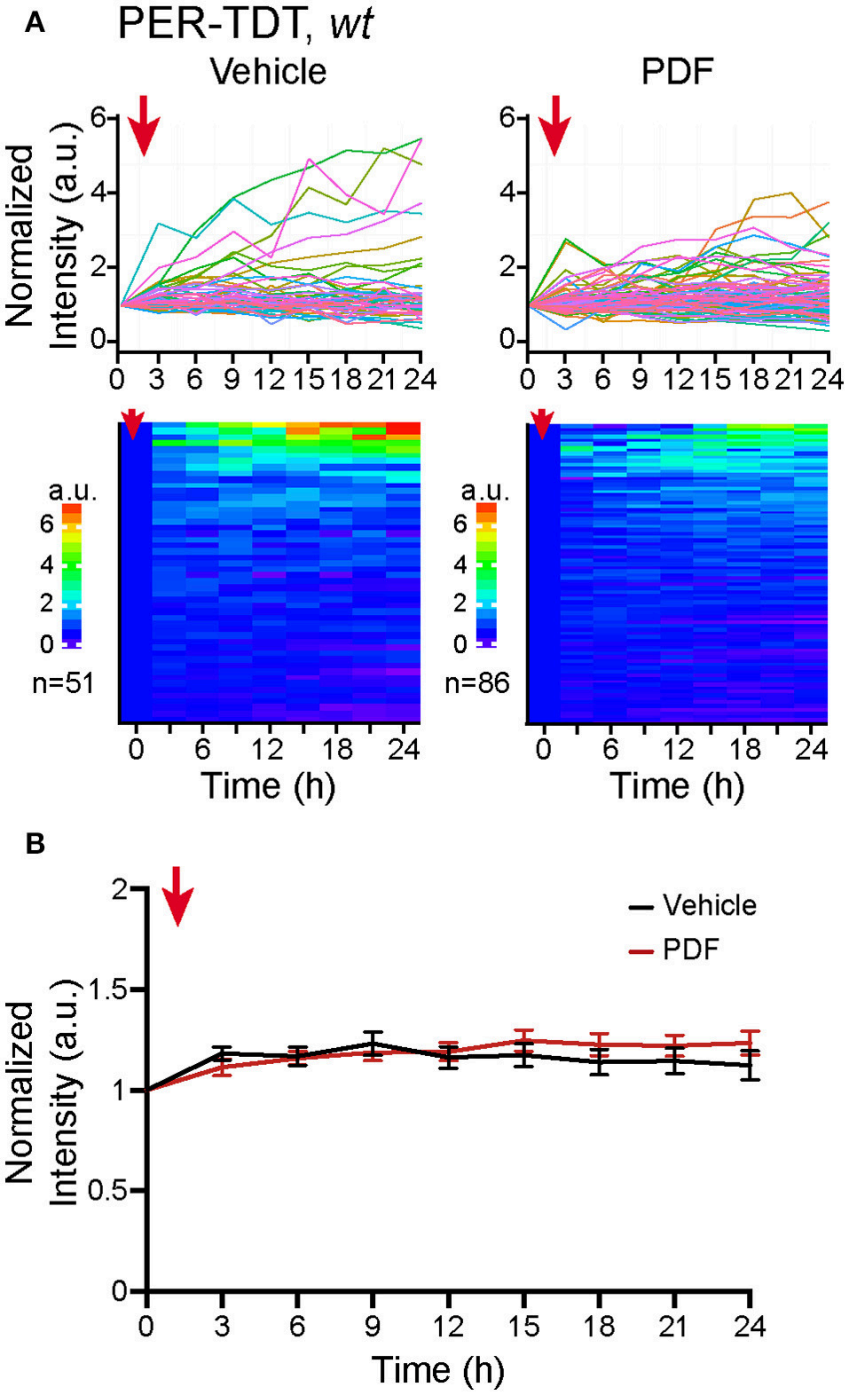
No upregulation of PER protein reporter expression *in vitro* following PDF application. **(A)** PER-TDT expression in *wt* dissociated cultured neurons with or without PDF treatment (2 μM), added at *t* = 0. Graphs and heatmaps show the relative fluorescence intensity of individual cells normalized to the value at *t* = 0. **(B)** Average PER-TDT levels ± SEM. Comparison between PDF- and vehicle-added groups by two-way ANOVA with Sidak's correction for multiple comparisons showed no significant differences between two groups. The red arrows indicate the time of drug application.

Therefore, we added 2 μM of PDF to the culture medium, a concentration sufficient to upregulate PER-TDT levels in brain explants (Sabado et al., 2017), and monitored PER-TDT expression every 3 h for subsequent 24 h. Unexpectedly, the addition of PDF did not affect PER-TDT expression patterns of dissociated clock neurons (Figures 3A,B). These results suggest that, in contrast to the effect of PDF on CLK/CYC-mediated transcription, stabilization of PER by PDF does not occur by default in individual clock neurons but requires them to be in an *in vivo*-like context.

**Figure 4:**
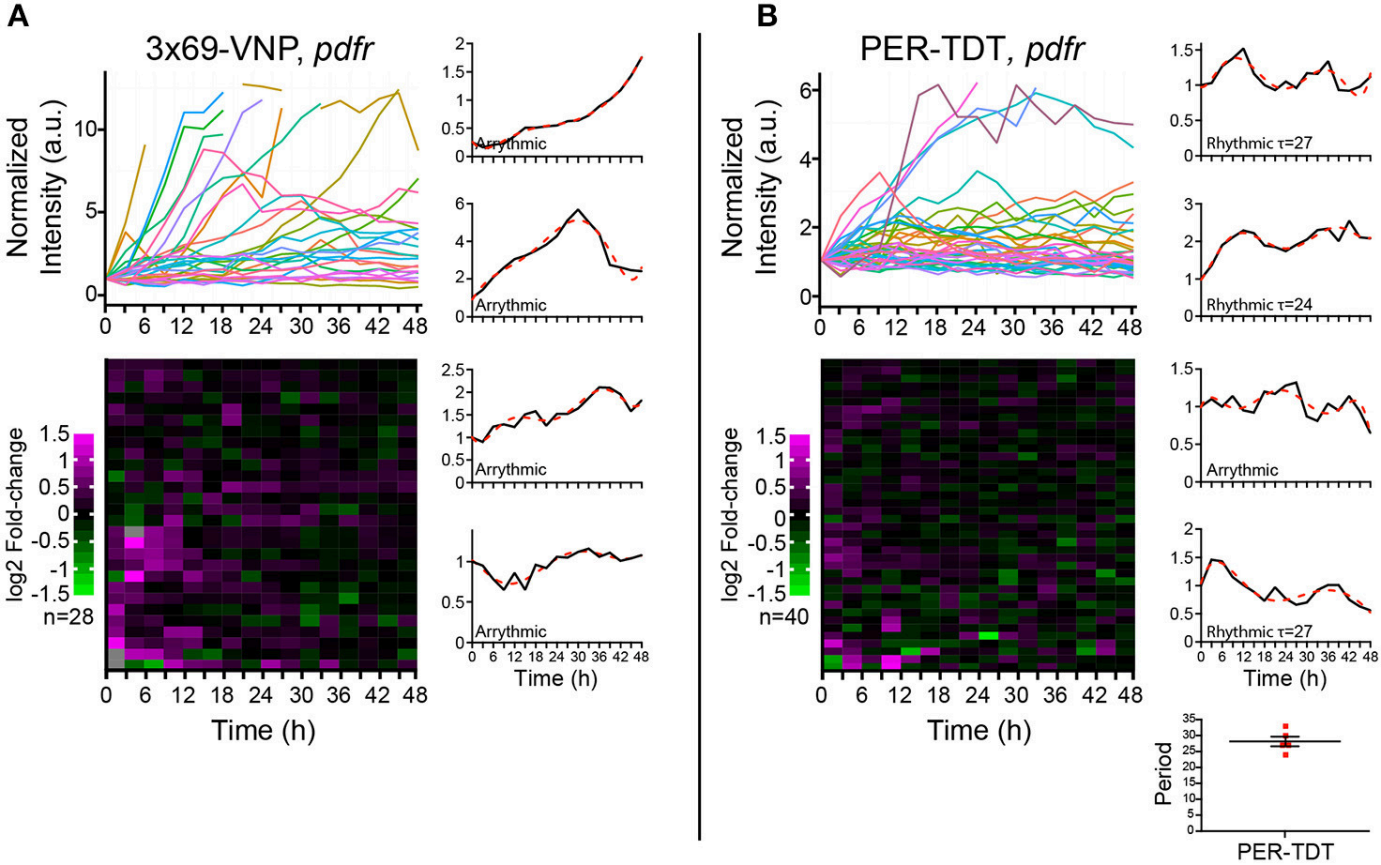
Properties of the *Drosophila* neuronal clocks in the absence of PDF signaling. **(A)** 3 × 69-VNP and **(B)** PER-TDT expression levels in dissociated cultured neurons prepared from *pdfr* mutants, normalized to the value at *t* = 0. Unlike in the *wild-type* culture, 3 × 69-VNP levels fluctuate in the *pdfr* mutant culture. Graphs and raster plots are as in Figure 1. Bottom right, periods of the neurons that showed circadian rhythms in PER-TDT levels (12.5% of PER-TDT-expressing cells). Mean period ± SEM was 28.2 ± 1.53 h. R, rhythmic; AR, arrhythmic.

PDF acts through the PDF receptor (PDFR) that belongs to the G protein-coupled receptor family (Hyun et al., 2005; Lear et al., 2005; Mertens et al., 2005). To learn more about the regulation of individual molecular clocks in dissociated neurons by PDF signaling, we next monitored the reporter expression in cultured clock neurons prepared from *pdfr* null mutants. In this culture, the medium should contain PDF released from the LNvs, whereas PDF/PDFR signaling is absent in clock neurons. PDF has an important presynaptic role in the development of the LNvs (Gorostiza and Ceriani, 2013), which is not affected in this condition. Interestingly, the majority of clock neurons of *pdfr* mutants did not show a steady increase but fluctuations in 3 × 69-VNP levels, although none were circadian (Figure 4A). This observation suggests that there is a persistent stochastic CLK/CYC-mediated transcription in isolated clock neurons in the absence of PDF/PDFR signaling, which gives rise to the fluctuating patterns of the 3 × 69-VNP signal. This finding also indicates that, in *wt* culture, PDF is present in the media and constantly upregulates CLK/CYC-mediated transcription, resulting in the steady increase of 3 × 69-VNP levels. The proportion of cells displaying PER-TDT rhythms was approximately the same between *wt* and *pdfr* (12.5%, 28.2 ± 1.53 h; Figure 4B). This result is concordant with the above finding that PER stability is insensitive to PDF signaling in dissociated neurons (Figures 3A,B).

## Discussion

Here, we examined the state of autonomy of *Drosophila* clock neurons by monitoring the expression of two circadian fluorescent reporters, the transcriptional reporter 3 × 69-VNP and PER protein reporter PET-TDT (Sabado et al., 2017), in long-term time-lapse imaging of dispersed clock neurons. Both reporters are expressed but in a non-circadian manner in most cells. 3 × 69-VNP levels exhibit a steady increase, whereas PER-TDT levels fluctuate and do not increase over time. While approximately one-tenth of clock neurons show PER-TDT level fluctuations with periods in the circadian range, no periodic nuclear translocation of PER-TDT was observed. These results suggest that *Drosophila* circadian clocks are poorly cell-autonomous.

### What underlies the loss of rhythms in dissociated clock neurons?

PER is the major repressor of the core TTFL of the molecular clock. Most clock neurons in culture show cytoplasmic enrichment of PER and TIM (Figures 2C–E), resembling the state of clock neurons around ZT14, a time when CLK/CYC-target gene transcription is at its maximum. Together with the finding that no periodic PER-TDT nuclear localization is observed in cultured clock neurons (Figure 2F), these results suggest that the feedback inhibition of CLK/CYC transcriptional activity by nuclear PER is impaired in isolated clock neurons, resulting in the constant upregulation of CLK/CYC-mediated transcription. Constant rise of 3 × 69-VNP levels in *wt* dissociated neurons (Figure 1B) fits this model. Importantly, 3 × 69-VNP levels exhibit fluctuations but not a steady increase in *pdfr* mutant cells in dispersed culture (Figure 4A). This observation rules out that the steady increase of 3 × 69-VNP levels in *wt* dissociated neurons is caused by a general impairment of protein degradation systems. Furthermore, it indicates that CLK/CYC-mediated transcription sustains in a stochastic manner in dissociated neurons even in the absence of PDF signaling.

Cytoplasmic enrichment and the absence of rhythmic nuclear accumulation of PER suggest an impairment in nuclear translocation or the stability of nuclear PER. PER nuclear entry is controlled by multiple kinases, including CK2 (Helfrich-Forster, 2005; Chiu et al., 2008), SGG (Martinek et al., 2001), and probably a still unknown proline-directed kinase (Ko et al., 2010). PER phosphorylation by DBT promotes PER degradation, which is prevented by binding to TIM (Kloss et al., 1998; Ko et al., 2002; Kim et al., 2007). Phosphorylated PER enters the nucleus as PER/TIM dimer, where dephosphorylation by PP1 (Fang et al., 2007) and PP2A (Sathyanarayanan et al., 2004) prevents the phosphorylation at the sites promoting the binding of the E3 ubiquitin ligase SLIMB and eventual degradation (Chiu et al., 2008). Therefore, some of these post-translational mechanisms are probably dysregulated in the dispersed clock neurons.

### Network-dependent effect of PDF signaling on the molecular clock

Our results also suggest that signaling downstream of PDF/PDFR may work differently in isolated clock neurons, which exacerbates the dampening of the molecular rhythms. In brain explants during the night, PDF enhances CLK/CYC-mediated transcription via neuronal activity-dependent but c-AMP independent mechanism. At the same time, PDF signaling stabilizes PER via cAMP-PKA dependent mechanism (Li et al., 2014; Seluzicki et al., 2014; Sabado et al., 2017). The latter mechanism seems to be inhibited in dispersed clock neurons, as the addition of PDF to the culture medium or *pdfr* mutation did not affect PER-TDT expression profile (Figures 3, 4B). Thus, PER is probably less stable in dissociated neurons even in *wt* culture than *in vivo*, which also impairs negative feedback on CLK/CYC-mediated transcription.

We previously showed that the addition of PDF in the medium increases the 3 × 69-VNP levels in dissociated neurons, whereas PDF upregulates 3 × 69-VNP levels only during the night in brain explants (Sabado et al., 2017). Collectively, these results postulate that transcriptional response to PDF is negatively regulated during the day in the brain. In contrast, our results here indicate that PER levels are insensitive to PDF signaling in dissociated culture, whereas PDF stabilizes PER during the night in brain explants. Therefore, the nighttime-specific effect of PDF on PER stabilization probably occurs via a positive regulatory mechanism that happens only within the intact circadian circuit. These negative and positive gating of molecular clock response to PDF signaling requires cycling clocks, which appear to be the emergent property of the intact circadian circuitry (Supplementary Figure 3). Intriguingly, recent studies using *in vivo* Ca^2+^ imaging demonstrated that PDF controls the “morning” and “evening” phases of the Ca^2+^ rhythms in different subpopulation of pacemaker neurons, independent of their molecular phases (Liang et al., 2016, 2017). Thus, our results add to a growing body of evidence that PDF differentially regulates molecular and neuronal rhythms through complex and network-dependent mechanisms.

Of note, unlike the l-LNvs, the s-LNvs do not express the neuropeptide amidation enzyme PHM; hence, the PDF released from the s-LNvs might be either non-amidated or amidated by another amidation machinery (Helfrich-Forster, 2009, Taghert et al., 2001). Since larval LNvs are the precursors of adult s-LNvs, we used non-amidated PDF for all the pharmacological experiments in this and our previous study (Sabado et al., 2017). Non-amidated PDF can trigger PDFR-dependent, time-of-day- and activity-dependent response of molecular clockwork in cultured larval brains as well as TTX-sensitive CLK/CYC-mediated transcription in cultured clock neurons (Sabado et al., 2017). Thus, although non-amidated neuropeptides have shorter half-lives and are less bioactive than the amidated ones, non-amidated PDF is bioactive at least in these assays. Nevertheless, since PDF secreted from larval LNvs and adult s-LNvs may as well be amidated (Taghert et al., 2001; Park et al., 2008), the results of these assays might not fully represent the native activity of endogenous PDF. It will be important in future studies to validate whether non-amidated PDF can activate PDFR and also test the effect of amidated PDF in the same assays.

### Network-driven molecular oscillations: a conserved feature of neuronal clocks?

While no cells in dissociated culture exhibit circadian rhythms in 3 × 69-VNP expression, approximately one-tenth of clock neurons display PER-TDT fluctuations with periods in the circadian range (Figures 1, 4). These results suggest that, certain post-transcriptional mechanisms involved in PER protein stability or transcriptional regulation via *per* regulatory sequences other than the CRS is still functioning in dispersed clock neurons. These regulatory mechanisms prevent over-accumulation of PER and even produce PER protein oscillations in the circadian range in some cells. Our results thus showcase the emerging notion that post-transcriptional controls reinforce robustness of circadian oscillation (Lim and Allada, 2013).

A previous work in mammals has shown that, in low-density dispersed culture, only ~18% of SCN neurons display circadian rhythms with periods ranging from 15 to 35 h (Webb et al., 2009). This is reminiscent of our observation that ~13% of isolated fly clock neurons exhibited PER protein rhythms. Moreover, it is noteworthy that we did not detect obvious cell-type specificity as to which neurons display circadian rhythms in dissociated culture. This implies that it is probabilistic rather than deterministic whether or not a neuron displays PER rhythms in isolation. Similarly, it was reported that isolated SCN neurons sustain or lose rhythmicity in random fashion (Webb et al., 2009). Thus, both *Drosophila* and SCN neurons seem to be poor oscillators that require network interactions to generate robust circadian rhythms. Whether or not a given pacemaker neuron sustain circadian molecular rhythms isolated from the intact circuit probably depends on the cell's physiological state, such as ion conductance and excitability. It has been proposed that the sensitivity of SCN neurons to the intercellular communication provides the circadian network the plasticity to promptly respond to changes in environmental conditions and produce a coherent output (Ko et al., 2010; Webb and Oates, 2016). It may also be a common principle of neuronal clocks in *Drosophila* and mammals.

## Materials and methods

### Fly strains

*Drosophila* were reared at 25°C on a corn-meal medium under 12:12 h light-dark cycles (LD). The *1982clk-gal4* line (Gummadova et al., 2009), The GAL4 enhancer trap line *gal1118* (Blanchardon et al., 2001), *cry*^*0*^ (Dolezelova et al., 2007), and *pdfr*^*5304*^ (Hyun et al., 2005), *UAS-mCD8::VENUS* and *UAS-mCD8::RFP*, 3 × 69-Venus and *per-TdT* lines (Sabado et al., 2017) have been previously characterized.

### Primary neuron culture

Brains of LD-entrained non-wandering L3 larvae were dissected in ice-cold saline solution (Jiang et al., 2005) at ZT11. Dissociated neuron culture was performed as previously described (Kuppers-Munther et al., 2004; Saad et al., 2012) with the following modifications. In brief, the dissected brains were enzymatically treated with 50 unit/mL papain (Worthington) and mechanically dissociated. The cell suspension was then plated on glass-bottom dishes (35 mm MatTek petri dish, 10 mm microwell with 0.16/0.19 mm coverglass) coated with Concanavalin A (Sigma). Approximately 3.1 ~ 4 × 10^5^ cells, which corresponds to the suspension derived from ca. Ten larval brains, were plated in each dish (4 ~ 5 × 10^5^ cells/cm^2^). Once the cells were attached to the glass bottom, the dish was flooded with SM^active^ (Supplementary Table 1). The dissociated neuron culture was incubated at 25°C in 80% relative humidity and in constant darkness for 2 days prior to time-lapse imaging. Time-lapse imaging was carried out in the same culture conditions.

For pharmacological experiments, the neuropeptide PDF (custom-made by Chi Scientific, H-NSELINSLLSLPKNMNDA-OH; 2 μM) or the vehicle (DMSO) was added to the cell culture medium just before the start of the time-lapse imaging.

### Immunostaining of cultured neurons

Anti-PER and Anti-TIM immunostaining of the cultured neurons was performed as previously described with some modifications (Nawathean et al., 2005). After 2 or 4 days in culture, the medium was discarded, and the culture was washed with phosphate-buffered saline (PBS); then, the cells were fixed with 1% paraformaldehyde in PBS for 20 min at room temperature. The cells were then washed with 0.1% Triton in PBS, and 1:1,000 rabbit anti-PER and 1:2,500 rat anti-TIM (gift from F. Rouyer) in blocking buffer were used as the primary antibodies. The secondary antibodies, anti-rabbit-Alexa-633 and anti-rat-Alexa-555 (Invitrogen), were diluted 1:500 in the blocking buffer.

### Live imaging

A Leica TCS SP5 tandem scanner confocal microscope was used for fluorescence imaging. The same parameter settings were applied to image all the samples of the same type. Immunostained cultured neurons were scanned using a × 63 water-immersion objective with the galvo scanner at 400 Hz. For time-lapse imaging, × 63 objective was used and the images were acquired every 3 h for 48 h using the resonant scanner at 8,000 Hz with high-sensitivity HyD detectors. The bi-directional scan was used together with 8 × line average. A Z-section step of 1.7 μm × 8 was used for imaging dissociated cultured neurons. A 514 nm laser was used to excite VENUS fluorophore (0.68 μW/cm^2^ to image VNP, 0.40 μW/cm^2^ for mCD8::VENUS), and a 561 nm laser was used for the TdTOMATO and mRFP fluorophores (14.8 μW/cm^2^ for PER-TDT, 5.71 μW/cm^2^ for mCD8::RFP). Laser intensity was measured at the level of the sample with a microscope slide power meter (Thorlabs, S170C).

### Image analysis

The fluorescence intensities in immunostained cultured neurons and the time-lapse movies of the cultured neurons were measured with the Imaris software (Bitplane). A 3D mask built from the fluorescent signal emitted by the clock neurons over the time course was created using the surface tool of Imaris. The same threshold settings, including background subtraction, were applied to all the samples of the same type. The intensity SUM was then extracted from the statistical data that were automatically generated by the program.

### Time series data analysis

Fluorescence intensity time series data were normalized to the value at *t* = 0 except for the raster plots for intensity fold-change. Heatmaps representing the fluorescence intensity time course were generated with an in-house R script. Each row represents the data of a single cell. Rows were ordered by the highest intensity over 24 h. To generate raster plots for intensity fold-change, the value at *t* = n + 1 was divided by the value of *t* = n, and the log2 of the fold-change value was plotted over time using an in-house R script. The log2 fold-change values were color-coded, in which positive fold-changes are shown with a magenta gradient, no change is shown in black, and negative changes are shown with a green gradient. The hierarchical clustering analysis (complete linkage method) was performed to find similar clusters and visualize the plots accordingly.

For rhythm analysis, a combination of manual inspection and Maximum Entropy Spectral Analysis (MESA) was used to detect rhythmicity and estimate the period. The data of the entire recording period (48 h) were subjected to the analysis. MESA analysis was performed using an in-house R script containing the spec.ar function. MESA was chosen because, unlike other methods, it is adapted for the detection of the rhythms in short or noisy time series (Dowse et al., 1989; Levine et al., 2002; Klarsfeld et al., 2003). First, the intensity time course plots were generated using Excel or Prism (GraphPad Prism version 6.0c for Mac, GraphPad Software, San Diego California USA, www.graphpad.com). Because we found that 6th-order polynomial regression models best fit the data, we superimposed the 6th-order polynomial trend lines to the graphs to facilitate the detection of rhythmicity. Then, the time series data that have circadian rhythms, defined by the peak-to-peak interval from 18 to 36 h, were identified by manual inspection. However, the polynomial trend lines were not used for determining the period. In parallel, the intensity time series data without normalization were analyzed with MESA without filtering. The time series data were scored as circadian only when both the manual and MESA analyses detected the rhythms with a period between 18 and 36 h.

### Statistical analysis

Statistical analyses were performed with GraphPadPrism software to compare the expression of antibodies or transgenes in different genetic backgrounds or pharmacological conditions. Non-parametric tests were used when the data did not follow a normal distribution. To compare the effect of one condition (genetic backgrounds or pharmacological conditions) parametric or non-parametric *T*-Tests or one-way ANOVA with a correction for multiple comparisons (depending on the number of data sets to compare) were used. For live imaging experiments, time was an added condition, hence, two-way ANOVA with a correction for multiple comparisons were utilized. Details of the used statistical tests are given in the figure legends.

## Author contributions

VS and EN designed the work. VS and LV performed the experiments and analyzed the data. VS, LV, and EN wrote the manuscript.

### Conflict of interest statement

The authors declare that the research was conducted in the absence of any commercial or financial relationships that could be construed as a potential conflict of interest.
