# Supplementary material for: Evaluating the Autonomy of the Drosophila Circadian Clock in Dissociated Neuronal Culture
Source: Front Cell Neurosci. 2017 Oct 12;11:317. doi: 10.3389/fncel.2017.00317 (PMC5643464; doi:10.3389/fncel.2017.00317)
Supplement: Supplementary file 3 [file DataSheet1.PDF]

*Supplementary Material*

# Evaluating the autonomy of the *Drosophila* circadian clock in dissociated neuronal culture

Virginie Sabado[1], Ludovic Vienne[1], and Emi Nagoshi[1,]*

*** Correspondence:** Emi Nagoshi: emi.nagoshi@unige.ch

## 1 Supplementary Data

Captions for Movies S1 and S2

**Movie S1**
3x69-VNP expression in cultured clock neurons labeled with *1982clk*-gal4, *UAS-mCD8::RFP*. Images were taken every 3 hr for 48 hr. Left and middle, 3x69-VNP is shown in green, and the clock-neuron marker is shown in magenta. Right, a 3D surface rendered movie of a clock neuron is shown with a rainbow scale.

**Movie S2**
PER-TDT expression in cultured clock neurons. Images were taken every 3 hr for 48 hr. Left, PER-TDT is shown in magenta. Right, a 3D surface rendered movie of a clock neuron is shown with a rainbow scale.

# 2 Supplementary Figures and Tables

## 2.1 Supplementary Figures

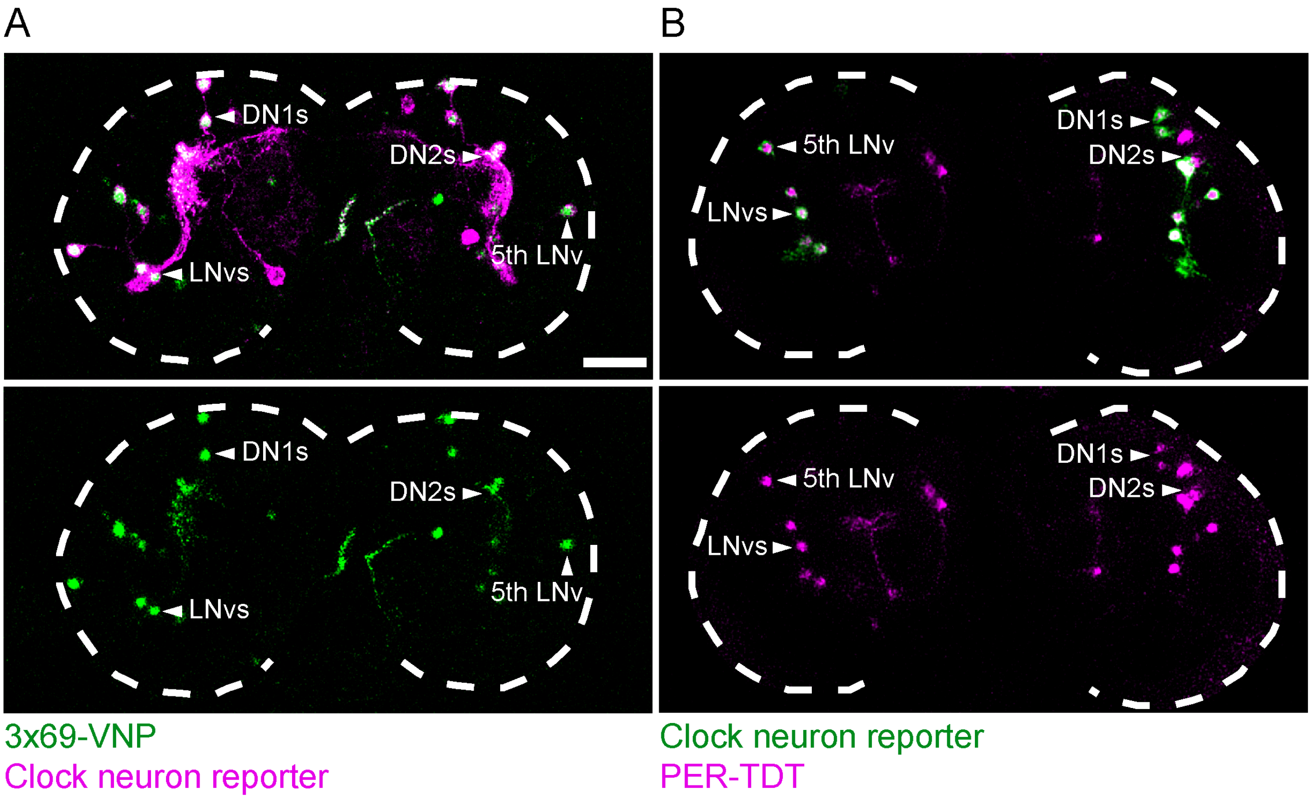


**Supplementary Figure 1. Expression of the fluorescent circadian reporters in the larval brain.** Representative confocal images of the brains of larvae expressing (A) 3x69-VNP or (B) PER-TDT reporters together with UAS-mCD8::RFP (A) or UAS-mCD8::Venus (B) driven by *1982clk-gal4*. Scale bar, 25 µm.

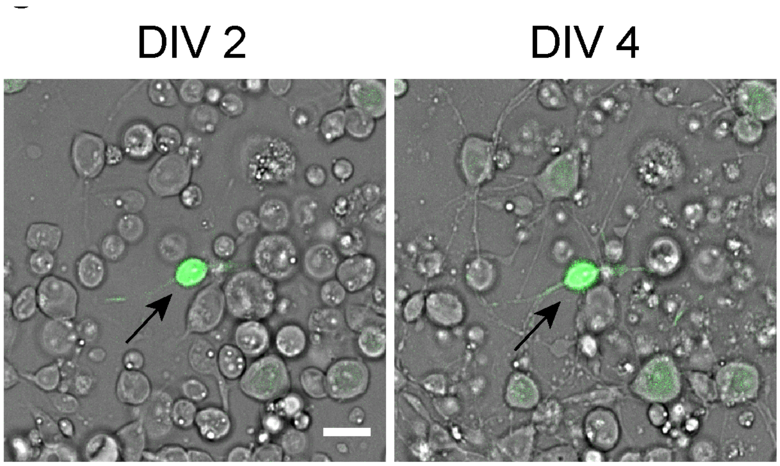


**Supplementary Figure 2. Dissociated cultured neurons derived from L3 larval brains.** Representative images of cultured neurons expressing 3x69-VNP (green) at the start (day 2 *in vitro*, DIV2) and the end (DIV4) of time-lapse imaging. Scale bar, 10 μm.

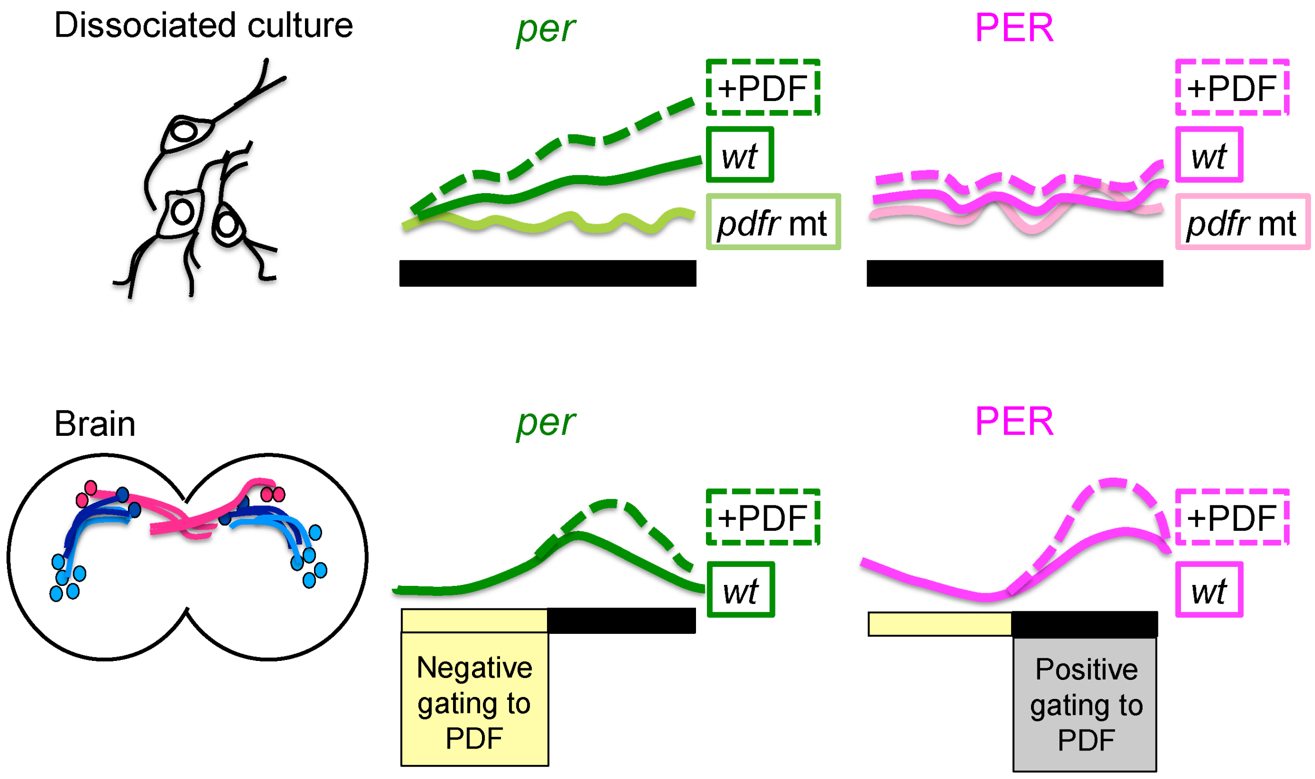


**Supplementary Figure 3. Effect of PDF on the molecular clocks *in vitro* and *ex vivo*.** In dissociated culture, PDF upregulates CLK/CYC-mediated transcription in clock neurons. Consequently, 3x69-VNP, which reports *per* mRNA expression, exhibits a steady increase in the presence of PDF signaling and shows fluctuations in its absence in *pdfr* mutants (top left). In isolated brains, PDF upregulates 3x69-VNP levels only during the night, suggesting that transcriptional response to PDF is suppressed during the daytime (bottom left). PER-TDT, and thus PER stability, is insensitive to PDF signaling in dissociated cultures (top right). In contrast, PDF upregulates PER-TDT levels during the night in brain explants, suggesting a nighttime-specific mechanism of PER stabilization by PDF (bottom right). The positive and negative gating to PDF occurs only in the cycling system, which is likely an emergent property of the intact circadian circuit.

## 2.2 Supplementary Tables

**Table 1. Preparation of SM$^{active}$ medium (1X) for *Drosophila* dissociated neuron culture**

| **Reagents** | **Concentration** |
|---|---|
| $KH_2PO_4$ | 4.18 mM |
| $CaCl_2$ | 1.05 mM |
| $MgSO_4.7H_2O$ | 0.7 mM |
| NaCl | 116 mM |
| $NaHCO_3$ | 0.7 mg/ml |
| Glucose | 2 mg/ml |
| Trehalose | 2 mg/ml |
| α-Ketoglutaric acid | 0.35 mg/ml |
| Fumaric acid | 60 µg/ml |
| Malic acid | 0.6 mg/ml |
| Succinic acid | 60 µg/ml |
| Yeast extract | 2 mg/ml |
| NON heat-inactivated FCS | 20% |
| $dH_2O$, autoclaved | |
| The solution was sterilized with a 0.22 µm filter and incubated at 25 °C in the dark for 72 h. Then the following reagents were added. | |
| Insulin | 2 µg/ml |
| Bis-Tris pH 6.8 | 5 mM |

The pH was adjusted to 6.8-6.9 by adding ~8 drops of NaOH (10 N). The solution was again sterilized with 0.22 µm filter, flushed frozen in $LN_2$ and stored at -80 °C.
